# Simultaneous and Dose Dependent Melanoma Cytotoxic and Immune Stimulatory Activity of Betulin

**DOI:** 10.1371/journal.pone.0118802

**Published:** 2015-03-10

**Authors:** Kathrin Pfarr, Corina Danciu, Olga Arlt, Christina Neske, Cristina Dehelean, Josef M. Pfeilschifter, Heinfried H. Radeke

**Affiliations:** 1 pharmazentrum frankfurt/ZAFES, Institute of General Pharmacology and Toxicology, Clinic of the Goethe University, Frankfurt/Main, Germany; 2 Departments of Pharmacognosy and Toxicology, Faculty of Pharmacy, University of Medicine and Pharmacy Victor Babes, Timisoara, Romania; Technische Universitaet Muenchen, GERMANY

## Abstract

Conventional cytostatic cancer treatments rarely result in the complete eradication of tumor cells. Therefore, new therapeutic strategies focus on antagonizing the immunosuppressive activity of established tumors. In particular, recent studies of antigen-loaded dendritic cells (DCs) eliciting a specific antitumor immune response has raised the hopes of achieving the complete elimination of tumor tissue. Genistein, fingolimod and betulin have already been described as active compounds in different types of cancer. Herein, we applied an integrated screening approach to characterize both their cytostatic and their immune-modulating properties side-by-side. As will be described in detail, our data confirmed that all three compounds exerted proapoptotic and antiproliferative activity in different B16 melanoma cell lines to a given extent, as revealed by an MTT assay, CFSE and DAPI staining. However, while genistein and fingolimod also affected the survival of primary bone marrow (BM) derived DCs of C57BL/6 mice, betulin exhibited a lower cytotoxicity for BMDCs in comparison to the melanoma cells. Moreover, we could show for the first time, that only betulin caused a simultaneous, highly specific immune-stimulating activity, as measured by the IL-12p70 release of Toll-like receptor 4-stimulated BMDCs by ELISA, which was due to increased IL-12p35 mRNA expression. Interestingly, the activation of DCs resulted in enhanced T lymphocyte stimulation, indicated by increased IL-2 and IFN-γ production of cytotoxic T cells in spleen cell co-culture assays which led to a decreased viability of B16 cells in an antigen specific model system. This may overcome the immunosuppressive environment of a tumor and destroy tumor cells more effectively in vivo if the immune response is specific targeted against the tumor tissue by antigen-loaded dendritic cells. In summary, cytostatic agents, such as betulin, that simultaneously exhibit immune stimulatory activity may serve as lead compounds and hold great promise as a novel approach for an integrated cancer therapy.

## Introduction

For decades the incidence of melanoma has been increasing, especially in the fair-skinned population. Malignant melanoma has gone from a rare disease into a cancer pathology with high medical importance. Although it is less frequent than other types of skin cancer, such as basal cell carcinoma or squamous cell carcinoma, melanoma represents the most dangerous form of skin cancer in clinical practice. Melanoma has a high death rate due to its prominent metastatic potential and its resistance to chemotherapy [1].

Given that overall cancer survival has not increased significantly during the past decades, new avenues of cancer treatment should focus on more than just the elimination of cancer cells by the induction of apoptosis or the inhibition of cell proliferation. In addition to surgery and chemotherapy, such as with dacarbazine, immunotherapy with interleukin 2 or interferon-α, has been used to treat malignant melanoma patients [2]. In general, the activation of immune cells and the tumor microenvironment are crucial for the control of tumorigenesis. For these reasons, efforts are ongoing to establish anti-cancer compounds that combine a cytotoxic activity against tumor cells with the ability to modulate the immune response [3]. Over one century ago, Paul Ehrlich performed trials with crude immune stimulating anti-cancer vaccination [4]; this indicated that the activation of dendritic cells (DC) via pattern recognition receptors (PRR) could be a useful tool to eliminate tumor tissue. Once the PRRs, e.g., Toll-like receptors (TLRs), are activated by exogenous or endogenous (“altered self”) danger signals (the so called pathogen-associated molecular patterns (PAMPs) and damage-associated molecular patterns (DAMPs)), the DCs migrate to the lymph nodes and present the processed antigen to the naive T cells [5]. Importantly, TLR4 and TLR2 have recently been shown to bind endogenous DAMPs, which are dispatched by stressed or dying cells. Thus, these two TLRs appear to be particularly critical for the orchestration of potentially therapeutic anti-cancer immune responses [6]. Similarly, we are interested in cytostatic compounds that potentially have co-stimulating effects on the TLR4/2 signaling pathways of DCs. For that reason, this study will investigate the influence of several drugs on the bone marrow (BM) derived DCs of C57BL/6 mice, which were stimulated with lipopolysaccharide (LPS) as a ligand for TLR4/2. This stimulation leads to the expression of IL-12 through NF-κB (nuclear factor 'kappa-light-chain-enhancer' of activated B-cells), IRF-3 (interferon regulatory factor 3) or AP-1 (activator protein-1) as well as to the activation of STAT4 (signal transducer and activator of transcription 4) [7]. Released IL-12 is essential for the generation of CD4^+^ type 1 T helper (Th1)- and CD8^+^ cytotoxic T cell (CTL)- mediated immunity to cancer. That is why IL-12 is considered to be a so called anti-tumorigenic cytokine. The protein IL-12p70 consists of two subunits, IL-12p35 and IL-12p40, whereas IL-12p40 can also assemble with another subunit, IL23p19, to form the pro-tumorigenic cytokine IL-23 [8]. The release of the anti-tumorigenic IL-12p70 heterodimer by activated DCs and the secretion of IFN-γ derived from activated Th1 cells promotes the activation of natural killer cells, macrophages and CD8^+^ CTLs. Tumor-specific CD4^+^ and CD8^+^ T cells migrate to the tumor site, and CTLs induce cell lysis and/or apoptosis in antigen-bearing tumor cells through the release of perforin and granzymes or by a Fas/Fas ligand interaction. Therefore, IL-12 is particularly responsible for the Th1- and CTL-driven anti-cancer immune response, which may also augment an increase of CTLs in combination with oxaliplatin [3].

DCs exhibit several characteristics that make them ideal as carriers of cellular tumor therapy. The first FDA-approved DC-based vaccine Sipuleucel-T (Provenge) is already in use for the treatment of prostate cancer. It stimulates the patient’s own immune system by treating isolated peripheral blood mononuclear cells with tumor antigens and transfused them back to the patient [9]. Human peripheral blood-derived DCs promote an anti-tumor CTL response also *in vitro* when prepulsed with melanoma-associated peptide epitopes [10]. In line with this, murine BMDCs promoted both prophylactic and therapeutic immunity against syngeneic tumors when first pulsed with tumor-derived peptides and subsequently applied *in vivo*.

Pursuing a bidirectional therapeutic mechanism, we investigated three candidates as anti-cancer drugs for melanoma: genistein, fingolimod and betulin. We have chosen these three plant derived, non-derivated parent compounds, which—with considerable likelihood—affect redox-sensitive or mitochondria-dependent signaling from TLRs to the IL-12 promoter. Specifically hints towards the TLR-mediated PAMP/DAMP-dependent IL-12 induction had been gathered from publications [11–13]. Additionally, all three candidates are published to have potential antiproliferative or proapoptotic activity in different cancer cell types [14–16]. This finally led to the selection of these three prototypical "mild" but comparable cytostatic compounds with at the same time, concomitantly highly differential effects on third signal generation by DCs.

The isoflavonoid genistein (GEN) is the aglycone of the heteroside genistin and represents the major active compound found in soybean, *Glycine max* (L.) Merr., *Fabaceae*. Previous studies have demonstrated that genistein possesses antioxidant, anthelmintic, anti-inflammatory, and antineoplastic properties [17,18,14]. Most importantly, genistein is already in clinical studies for patients with breast, prostate, bladder, kidney, pancreatic, and endometrial cancer (www.clinicaltrials.gov) because of its diversified antiproliferative effects [19].

Fingolimod (FIN) is a synthetic analog of the natural compound myriocin, a metabolite of the fungus *Isaria sinclairii* (Berk.) Lloyd, *Clavicipitaceae*. According to current theories, fingolimod exerts its immunosuppressive activity as a sphingosine-1-phosphate receptor modulator, causing the sequestration of lymphocytes in the lymph nodes and thereby preventing them from contributing to an autoimmune reaction [20]. In September 2010, the FDA approved fingolimod as the first oral drug for the treatment of relapsing forms of multiple sclerosis. Some studies have also described this agent as having cytotoxic activity against different types of cancer. Fingolimod induced necrotic cell death and autophagy in human ovarian cancer cells, reduced splenomegaly in leukemic mice and suppressed liver tumor metastasis in a rat liver tumor model by reducing the circulating endothelial progenitor cells [21,22,15].

The third substance we will focus on is betulin (BET). This pentacyclic triterpene is the precursor of betulinic acid (BA), which has been extensively studied as a selective treatment for human melanoma [23]. BA directly triggers mitochondrial membrane permeabilization, which is a central event in the intrinsic apoptotic process. It also affects Bcl-2 family members. In contrast to its potent cytotoxicity against cancer, non-neoplastic cells and healthy tissue remain relatively resistant to BA [24]. The precursor of BA, betulin, has been less well studied than its carboxylic form. Betulin exhibited inhibitory activity against A431, HeLa and MCF-7 cell lines and showed anti-angiogenic activity, which was determined by a clear reduction of blood vessel formation [16]. It was also active in skin cancerogenesis, as reported in a model of chemically induced cutaneous carcinoma [25]. Further investigations revealed its immune-modulating activity towards fibrotic liver stellate cells [26].

In the present study we simultaneously determined both the cytotoxic actions of genistein, fingolimod and betulin on melanoma cells as well as their immune modulating effects on dendritic cells and T cells. Interestingly, betulin showed simultaneous anti-melanoma and immune-stimulating activity in parallel, qualifying this compound for an integrated tumor cytotoxic and cellular immune therapeutic approach.

## Materials and Methods

### Substances

Genistein (4’,5,7-trihydroxyisoflavone), betulin (lup-20(29)-ene-3β,28-diol) and betulinic acid ((3β)-3-Hydroxy-lup-20(29)-en-28-oic acid) were obtained from Extrasynthese, Lyon, France, and fingolimod (FTY720; 2-amino-2-[2-(4-octylphenyl)ethyl]propane-1,3-diol) was kindly donated by V. Brinkmann, from Novartis Pharma AG, Basel, Switzerland. Betulinic acid derivatives B-10 and NVX-270 were kindly provided by Prof S. Fulda, Institute for Experimental Cancer Research in Pediatrics, Goethe University Frankfurt/Main. Roswell Park Memorial Institute (RPMI) 1640 medium with Glutamax, Dulbecco’s Modified Eagle’s Medium (DMEM), phosphate-buffered saline (PBS), penicillin, streptomycin, 4-(2-hydroxyethyl)-1-piperazine ethanesulfonic acid (HEPES) and trypsin were purchased from Gibco, Karlsruhe, Germany. Fetal calf serum (FCS), LPS, ethylene diaminetetraacetic acid (EDTA) and dimethyl sulfoxide (DMSO) were purchased from Sigma Aldrich, Munich, Germany. DMSO was used as a control and to prepare 10 mM stock solutions of all of the tested substances. The highest concentration of DMSO in the medium never exceeded 0.1% and did not influence any of the tested aspects in all of the experiments presented.

### Mice

Female C57BL/6 mice were used for the spleen or bone marrow isolation at the age of 8–20 weeks. C57BL/6 wild type mice were obtained from Janvier, France, and were maintained according to the institutional guidelines in a local animal facility, ZFE Frankfurt/Main, by Dr. A. Theisen. GO-Tg-TCR-Ova-1 (OT-I) mice expressing ovalbumin-specific transgenic T cell receptors (OVA TCR) were kindly provided by S. Sudowe, Dermatology, Mainz. OT I RAG mice express exclusively OVA TCR because they are additionally crossed with RAG1 (recombination activating gene 1) knockout mice. The OT I RAG mice were kindly provided by P. Knolle, Clinic of the University, Bonn. Mice were anesthetized with isofluran and killed by cervical dislocation without suffering. Afterwards, we dissected the respective organs and prepared single cell suspensions. There are no in vivo experiments included in this study. The researchers in charge of the experiments are authorized to breed, house, and sacrifice animals. Prior to this investigation we had approached the Goethe University animal welfare body and approval was waived for this study (Art. 4(3) in conjunction with Art. 7(2) sentence 3 German animal welfare law).

### Isolation of primary murine bone marrow-derived dendritic cells

Bone marrow-derived dendritic cells (BMDCs) were generated as described previously [27]. Briefly, the BM cells were collected from the femora and tibiae of the C57BL/6 mice. The bone marrow cells were depleted of red blood cells with lysis buffer and plated in DC culture medium (RPMI 1640 medium with Glutamax supplemented with 40 ng/ml granulocyte macrophage colony stimulating factor (GM-CSF), 10% FCS, 1% HEPES, 1 mM sodium pyruvate, 100 μM 2-mercaptoethanol, 100 unit/ml penicillin and 100 μg/ml streptomycin) in a humidified 5% CO_2_ incubator at 37°C. On day 4, two-thirds of the media was removed, and fresh media that contained additional GM-CSF (40 ng/ml) was added. On day 7, the non-adherent and adherent DCs were harvested. The DCs generated in this manner displayed typical morphologic features and exhibited surface markers as described for immature DCs. The BMDCs were seeded in well plates in serum free media with the density of the cells depending on the respective experiments described below. The stimulation of the cells was performed 1 h after seeding.

### Isolation of primary murine spleen cells

Spleen tissue of the OT-I mice was shredded and passed through 70-μm nylon pore size cell strainers (BD Falcon, USA). The red blood cells were destroyed with lysis buffer. The cells were washed and resuspended in complete Iscove basal medium (Biochrom AG, Berlin, Germany) that was supplemented with 100 U/ml penicillin, 100 μg/ml streptomycin, 2 mM L-glutamine, 1 mM sodium pyruvate, 100 μM non-essential amino acids, 0,1% ß-mercaptoethanol and 5% FCS for maintenance culture. The cells were counted and used appropriately.

### B16 melanoma cells

The B164A5 cells were acquired from Sigma Aldrich (ECACC, origin Japan, stored UK), and the B16F10 cells (ATCC CRL-6475) were acquired from the Naval Biosciences Laboratory, Manassas, USA, kindly provided by W. H. Böhncke, dermatology, Goethe University Frankfurt/Main. The B16OVA cells express SIINFEKL as antigen providing OVA peptide-loaded MHC class I molecules (H2kb) at their surface and were kindly provided by K. Reuter, University Medical Center, Mainz. All cell lines have a C57BL/6 background. B164A5 is a melanoma cell line derived from the skin of a C57BL/6 mouse strain, showing fibroblast-like characteristics and producing melanin. B16F10 is a subline which was derived from the parent B16 line by selection for their ability to form lung colonies *in vivo* after intravenous injection; they were subsequently established *in vitro* after 10 (F10) cycles of lung colony formation [28]. The B16 cells were cultured at 37°C in a 5% CO_2_ atmosphere in DMEM, supplemented with 10% FCS, 100 U/ml penicillin, 100 μg/ml streptomycin and 2% HEPES.

### Tumor cell proliferation assays

#### MTT (3-(4,5-Dimethylthiazol-2-yl)-2,5-diphenyltetrazolium bromide) proliferation assay

A MTT kit was acquired from Roche Applied Science, Mannheim, Germany. B16 cells (6x10^3^) were seeded in a 96 well microplate. After 24 h, a new medium containing 1% FCS and different concentrations of the active compounds genistein, fingolimod or betulin (150, 100, 50, 30, 15, 5, 1, and 0 μM) were added. On day three, the MTT reagent was added for 4 h and was converted by the mitochondrial reductase. The resulting purple crystals were dissolved in solubilization buffer and spectrophotometrically analyzed at 570 nm, using a reference of 656 nm in a microplate reader (SpectraMax M5e). The inhibition index of proliferation was calculated as 1—absorbance sample X / absorbance sample blank.

#### Trypan blue viability assay

B16 cells (5x10^5^) were seeded in 6-well plates for 24 h. A medium containing 1% FCS and different concentrations of the active compounds genistein, fingolimod or betulin (150, 100, 50, 30, 15, 5, 1, and 0 μM) was added. After 24 h, the cells were stained with trypan blue (Bio-Rad) and the amount of living cells was determined using a Bio Rad TC10 Automat Cell Counter. Trypan blue dye stains dead cells blue as described elsewhere [29].

#### CFSE (carboxyfluoresceindiacetate succinimidyl ester) proliferation assay

B16 cells, 5x10^6^ cells/ml, were washed with PBS, stained with 20 μM CFSE (TEFLabs, Inc., Austin, Texas, USA) and protected from light for 10 min. B16 cells (5x10^5^) were seeded in 6-well plates for 24 h in medium containing 1% FCS and different concentrations of the active compounds genistein, fingolimod or betulin (150, 100, 50, 30, 15, 5, 1, and 0 μM). Samples of the unstained and stained cells from day 0 to 4 were collected after 0 h, 24 h, 48 h, 72 h, and 96 h and fixed in a PBS buffer containing 1% FCS, 0.1% NaN_3_ and 1% paraformaldehyde. The samples were analyzed by FACS in the FL1 fluorescence channel using a BD Canto II FACS DIVA device. Flow Jo soft (7.6.5) was used for the data analysis.

#### Cell cycle analysis

A Cell Cycle Kit was purchased from Gene Script, Piscataway, USA. B16 cells (3x10^5^) were cultivated in 6-well plates for 24 h. Afterwards, the cells were incubated in medium containing 1% FCS and 100 μM genistein, 5 μM fingolimod or 5 μM betulin. Untreated cells served as the control. After 72 h, the cells were harvested and fixed with 70% ice cold ethanol at 4°C overnight. On the second day, the cells were incubated at 37°C for 30 minutes with RNase A and incubated at 4°C for 30 minutes with propidium iodide (PI) and protected from light. The samples were analyzed posterior in a FACS FL2 fluorescence channel using a BD Canto II (BD Biosciences, Heidelberg, Germany) FACS DIVA device. Flow Jo soft (7.6.5) was used for the data analysis.

### Determination of the pro-apoptotic activity of the compounds

#### Annexin V-FITC staining

The B16 cells and the BMDCs were cultivated for 24 h with appropriate concentrations of the different active compounds. BMDCs were gated as CD11c^+^ cells using a CD11c-PE tagged antibody (MCA 1369, AbD serotec). Translocation of the phosphatidylserine residues of the B16 cells and CD11c^+^ BMDCs were measured by incubating 10^6^ cells with 5 μl of annexin V-fluorescein (ImmunoTools GmbH, Oldenburg, Germany) and 5μl of 7-Amino-actinomycin D (7-AAD) (eBioscience, Germany) in a scheduled buffer for 15 min. The cells were analyzed by flow cytometry on FACS Canto II (BD Biosciences, Heidelberg, Germany) using a FACS DIVA device. The DMSO treated cells were used as a negative control, and the cells treated with 500 nM staurosporine (LC Laboratories, Woburn, USA) were used for the compensations as a positive control. Flow Jo soft (7.6.5) was used for the data analysis and presentation.

#### DAPI (4, 6-diamidino-2-phenylindole) staining

B16 cells (5x10^4^) were seeded in an 8-well chamber slide system (Thermo Scientific, NY, USA). After 24 h, the cells were incubated for 72 h in medium containing 1% FCS and 100 μM genistein, 5 μM fingolimod or 5 μM betulin, respectively. The cells were incubated for 5 minutes with 2 μg/ml DAPI (Roche Applied Science, Mannheim, Germany). Images of the stained nuclei of the cells were taken by an inverted florescence microscope system (Axiovert 200, Zeiss, Germany).

#### Western blot detection of Bax, Bcl-2, Caspase 3 and PARP proteins

The B16 cells were incubated in medium containing 1% FCS and 100 μM genistein, 5 μM fingolimod or 5 μM betulin for 72 h. The proteins were isolated using the BCA kit from Thermo Scientific, Rockford, USA, with albumin as the standard. An appropriate amount of cell lysates (15–50 μg protein) were resolved in 12.5% sodium dodecyl sulfate polyacrylamide gel and then transferred to a nitrocellulose membrane. The blots were blocked with 5% non-fat dry milk (for caspase 3 detection, acquired from Cell Signaling Technology, Danvers, USA) or 5% BSA (for Bax, Bcl-2 and PARP detection, acquired from Santa Cruz Biotechnology, USA) and probed using appropriate primary antibodies (Cell Signaling Technology, Danvers, USA) in a blocking buffer overnight at 4°C. The membrane was then incubated with the secondary antibody (donkey anti-rabbit, GE Healthcare, UK) conjugated with horseradish peroxidase (HRP) followed by detection using a chemiluminescence ECL kit (Thermo Scientific, Rockford, USA). To ensure equal protein loading, the membrane was stripped and reprobed with anti-β-actin antibody (Miltenyi Biotec GmbH, Gladbach, Germany). The blots were analyzed densitometrically by the use of the program Quantity One.

### Determination of the immune modulating activity of anti-cancer compounds

#### Cytokine measurement

Immature BMDCs (1x10^6^) were seeded serum free in 12-well plates and stimulated with 1 μg/ml LPS and appropriate concentrations of the active compounds for 20 h. The levels of IL-12p70, IL-6 and IL-10 in the supernatants were measured using duo set enzyme-linked immune sorbent assay (ELISA) development kits purchased from R&D Systems, USA. An ELISA kit from eBioscience, San Diego, USA was employed for the detection of the secreted IL-23.

OT I spleen cells (1.5x10^6^) were seeded serum free in 12-well plates and stimulated with 1 μg/ml LPS as well as with 5 μM genistein, 5 μM fingolimod or 5 μM betulin for 44 h. 13.5 μg/ml ovalbumin was added to the cell suspension to serve as an antigen. The T cell activity was determined by IFN-γ and IL-2 cytokine detection and measured in the collected supernatant by ELISA kits that were purchased from R&D systems, USA.

#### Real-time quantitative PCR analysis of cytokine mRNA

The cytokine mRNA expression was measured after stimulating 2x10^6^ immature BMDCs in a 6-well plate with 1 μg/ml LPS and 5 μM of the active compounds for 6 h. The RNA was isolated using an RNA isolation kit (PeqLab Biotechnologie GmbH, Erlangen, Germany) and was reverse transcribed to cDNA, which was then used for a real-time polymerase chain reaction (PCR). The primers and probes of the target genes were Taq-Man Assay-on-Demand Gene expression reagents (Applied Biosystems, Foster, USA): IL-12p35 (Mm00434165_m1) and IL-12p40 (Mm01288993_m1). GAPDH (Mm99999915_g1) was used as a control housekeeping gene. Relative expression was calculated using the ΔCT method.

#### Cytotoxicity assay of B16OVA cells

B16OVA cells were stained with 10 μM CFSE for 15 min at 37°C and were seeded in a 12-well plate (1x10^5^). 50 ng/ml IFN-γ was used to induce the MHC I expression on these melanoma cells. After 24 h, spleen cells (3.2x10^6^) isolated of wild type or OT I RAG mice were added. These spleen cells were pretreated with 1 μg/ml SIINFEKL and 50 Units/ml IL-2 or 5 μM betulin or both for 24 h. Moreover, the spleen cells were stained with Antibodies against CD11c, H2kb bound to SIINFEKL, CD3, CD8 and OVA TCR to determine the frequency of SIINFEKL-loaded dendritic cells as well as SIINFEKL-specific T cells within the spleen cell pool by FACS measurements. After 24 h the adherent B16OVA cells were collected, washed and measured by FACS analysis.

#### Statistics

The Prism software package (Graph Pad Prism 5.0 for Windows) was used for data collection and presentation. The data ranged from three to twelve separate experiments and is presented as the mean ± standard deviation (SD). All data presented resulted from independently repeated experiments. The number of repeats is given in each figure legend. An unpaired student t test, one-way ANOVA or two-way ANOVA followed by a Bonferroni post-test were used to determine the significant differences between the various experimental and control groups. *, ** and *** indicate p<0.05, p<0.01 and p<0.001, respectively, compared to the untreated or DMSO treated control group.

## Results

### Antiproliferative effects of genistein, fingolimod and betulin on B16 melanoma cell lines

**Fig 1:**
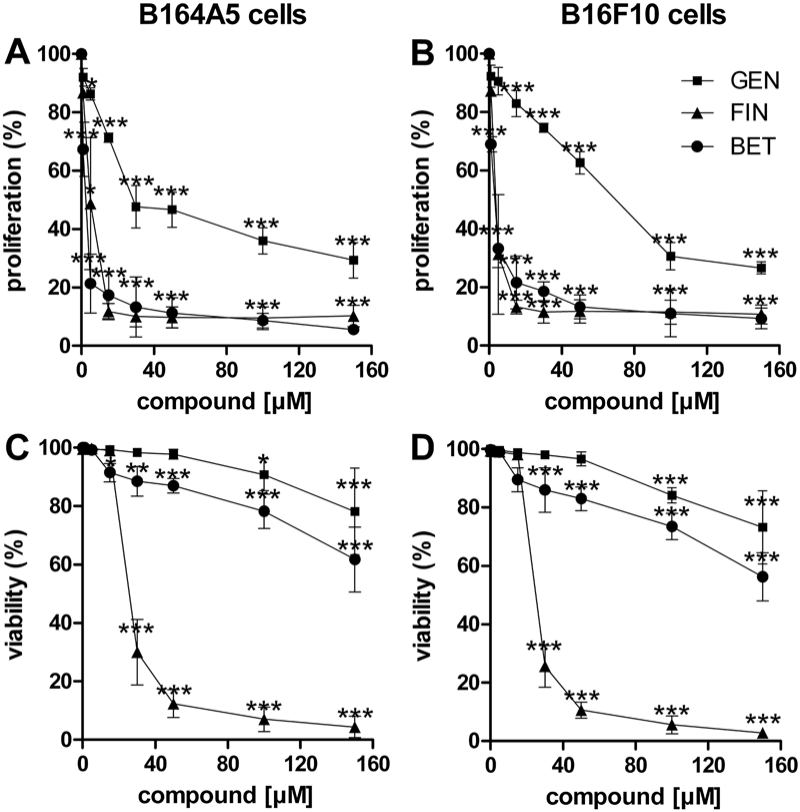
Dose-dependent effects of cytostatic drug treatment on B16 cell proliferation and viability. Proliferation was determined by an MTT assay (mean ± SD, n = 4) for genistein (GEN, square), fingolimod (FIN, triangle) and betulin (BET, circle) in the B16 melanoma cell lines. B164A5 (A) and B16F10 (B) cells were treated at concentrations ranging from 1 to 150 μM for 24 h and stained with the MTT reagent for 4 h. Additionally, the percentage of living cells was tested by trypan blue exclusion (mean ± D, n = 5) for the B164A5 (C) and B16F10 (D) melanoma cell lines. The cells were treated as described above and stained with trypan blue after 24 h. Significant differences compared to the untreated control cells were calculated by two-way ANOVA with a Bonferroni post-test.

The first approach to elucidate the cytotoxic mechanism of these three compounds was carried out by an MTT proliferation assay. Genistein, fingolimod, and betulin all dose-dependently reduced the activity of the mitochondrial oxidoreductase of the B16 cells (Fig. 1A, B). Both of the murine melanoma cell lines, B164A5 and B16F10, were affected in nearly the same range. An increasing concentration of genistein decreased the B16 cell viability to a lower extent than the other two substances. At the highest tested concentration of genistein, the cell viability was still 30% (p<0.001). In contrast, a concentration of only 15 μM of fingolimod or betulin was sufficient to suppress the proliferation rate of both melanoma cell lines to less than 20% (p<0.001). These differences were also reflected by the IC_50_ values calculated (Table 1).

**Table 1:** IC_50_ values of genistein, fingolimod and betulin in B16 melanoma cell lines.

Substance	Cell line	IC_50_ [μM]
**Genistein**	**B164A5**	**41.1**
	**B16F10**	**64.1**
**Fingolimod**	**B164A5**	**4.5**
	**B16F10**	**3.4**
**Betulin**	**B164A5**	**1.9**
	**B16F10**	**2.5**

IC_50_ values evaluated by the MTT assay for genistein, fingolimod and betulin in the B16 melanoma cell lines. The B16F10 and B164A5 cell lines were treated with various concentrations of genistein, fingolimod or betulin (0–150 μM) for 24 h and stained with MTT reagent for 4 h.

**Fig 2:**
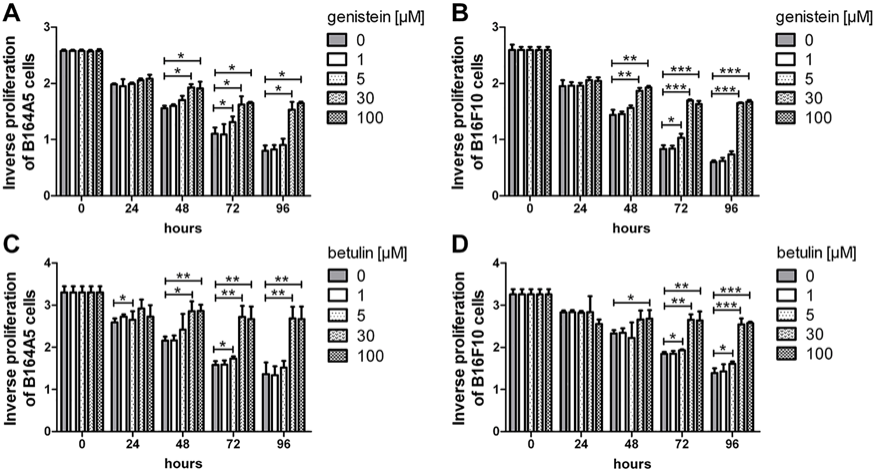
Time- and dose-dependent effects of cytostatic drug treatment on B16 cell proliferation. The decreasing intensity of the CFSE dye as a degree for the proliferation rate of the cells is shown (mean ± SD, n = 3). B164A5 (A, C) and B16F10 (B, D) melanoma cells were stained with CFSE and treated for 4 days with different concentrations of genistein (A, B) or betulin (C, D), respectively. Subsequent flow cytometry analysis of this cytosolic dye dilution as an indicator of frequency and number of cell divisions was determined.

To confirm these results, we measured the cytotoxic effect of the compounds by exclusion dye staining with trypan blue after 24 h (Fig. 1C, D). In line with the MTT data, the cell viability of the genistein-treated samples remained relatively high. Even at the highest concentration of genistein tested, over 70% of the cells were still alive (p<0.001). However, fingolimod displayed a similar high toxicity in the trypan blue staining as in the MTT assay. After stimulation of the cells with 50 μM fingolimod, over 90% of the cells were stained blue (p<0.001). In contrast with the MTT assay, the B16 cells treated with 150 μM betulin demonstrated a trypan blue exclusion capacity of 60% (p<0.01), whereas in the MTT test these cells had shown almost no mitochondrial activity starting from a concentration as low as 15 μM. As will be discussed, this finding may indicate an arrest in proliferation without the actual killing of the cells. To gather more information about these differences, a four day study was performed to characterize the cell division rate of the treated melanoma cells in more detail using the fluorescent dye CFSE. Because treatment of the cells with fingolimod led to a very efficient and fast cell death, the CFSE assay was performed for genistein and betulin treated cells only (Fig. 2). After 24 h, the proliferation of the B164A5 cells was already decreased when treated with 30 μM genistein (p<0.05). In contrast, 50 μM betulin was necessary for the same effect within the same time frame. Additionally, a low concentration of 5 μM genistein inhibited the proliferation rate of the B164A5 cells after 48 h, whereas 5 μM betulin needed 72 h to decrease the cell division rate (p<0.05). These two results again indicated the delayed antiproliferative effect of betulin. Over a longer period of time and in higher concentrations starting from 100 μM, betulin seemed to be a stronger antiproliferative agent than genistein. In the B16F10 cells, however, the antiproliferative response of betulin was weaker.

**Fig 3:**
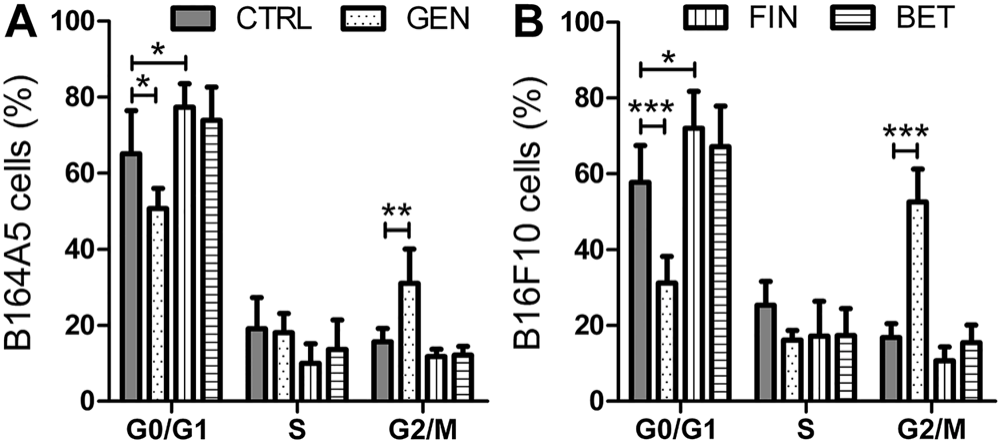
Effect of cytostatic drug treatment on the cell cycle phases of B16 melanoma cells. Cell cycle analysis of B164A5 (A) and B16F10 (B) melanoma cells treated with 100 μM genistein (GEN), 5 μM fingolimod (FIN) or 5 μM betulin (BET) for 72 h, respectively. The quantification of cells in G0/G1, S and G2/M were analyzed using flow cytometry by PI staining after 24 h (mean ± SD, n = 4). Significance was calculated using two-way ANOVA with a Bonferroni post-test.

To further differentiate the mechanisms of genistein, fingolimod and betulin, we analyzed the cell cycle phases. The concentration of the substances for this assay (100 μM genistein, 5 μM fingolimod, 5 μM betulin) were chosen based on the findings of the MTT assay compared to the apoptosis measurements described below. We assumed that the cytostatic effect is worth to get investigated at its best when the compound has relatively low apoptotic potential but high antiproliferative activity. This is true for 100 μM genistein as well as for 5 μM fingolimod and 5 μM betulin (S1 Fig.). To analyze the cell cycle phases we treated the B16 cells with the compounds for 72 h and stained them with PI (Fig. 3; S2 Fig.). High concentrations of genistein stopped the proliferation by blocking the cells during the G2/M phase (p<0.001). Fingolimod arrested the proliferation of the B16 cells by blocking the G0/G1 phase (p<0.05). Under the conditions described for both anti-melanoma compounds, the cell cycle arresting effect was more pronounced for the high metastatic cell line B16F10 compared to the low metastatic parent cell line B164A5. In contrast, betulin failed to target a specific step of the cell cycle at the concentration tested in this study.

### Pro-apoptotic effects of genistein, fingolimod and betulin on B16 melanoma cell lines and bone marrow derived dendritic cells (BMDCs)

**Fig 4:**
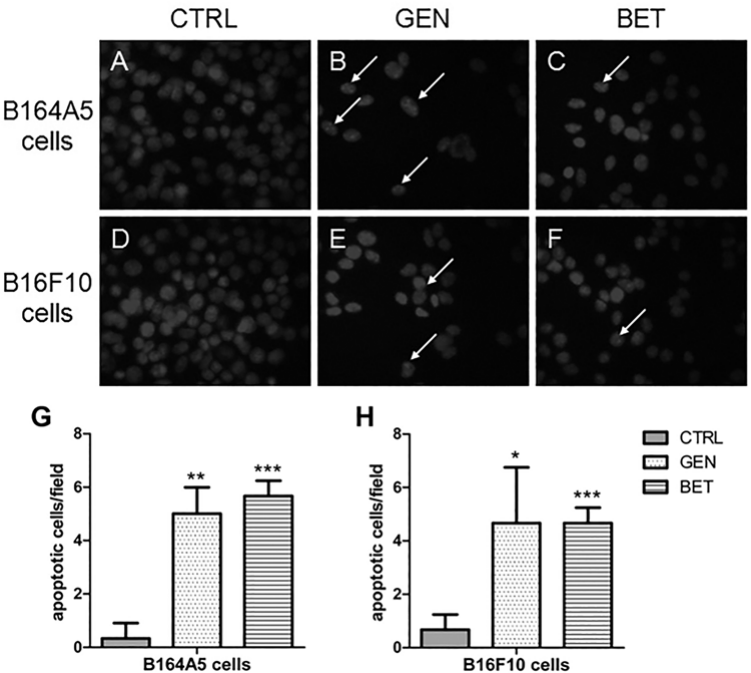
DAPI staining of B16 melanoma cells after treatment with cytostatic drugs. Representative images of DAPI-stained B164A5 (A–C) and B16F10 (D–F) cells after treatment with DMSO (A, D), 100 μM genistein (GEN: B, E) or 5 μM betulin (BET: C, F) for 48 h, respectively. Arrows indicate nucleus fragmentation. Apoptotic cells per field of three independently repeated experiments are counted and shown in bar graphs for the B164A5 (G) and B16F10 (H) cell line.

To elucidate the mechanisms of the antiproliferative activities and to detect the first signs of apoptosis, DAPI staining was performed after 48 h (Fig. 4). For both B16 cell lines, a reduction of the cell number and an increase in nuclear fragmentation compared to the control was recorded when the cells were incubated with 100 μM genistein or 5 μM betulin.

**Fig 5:**
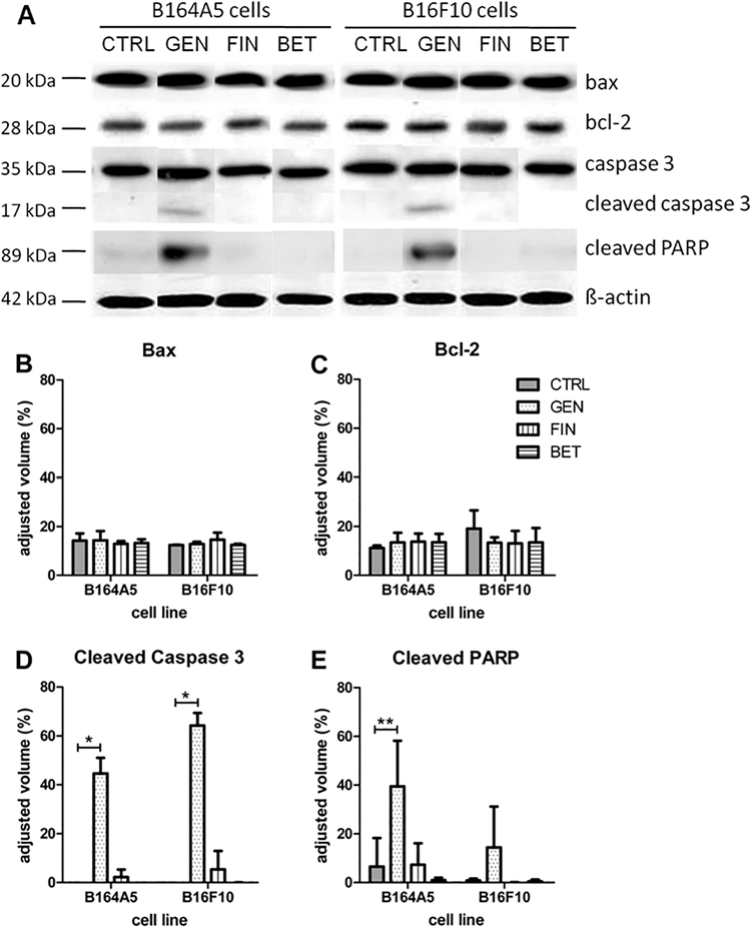
Analysis of proapoptotic and antiapoptotic molecules in B16 melanoma cells after treatment with cytostatic drugs. Representative Western blot results (A) as well as densitometric analysis of performed Western blots for Bax (B), Bcl-2 (C), cleaved caspase 3 (D) as well as cleaved PARP (E) expression in B16 melanoma cells after treatment with genistein, betulin or fingolimod for 72 h, respectively (mean ± SD, n = 3). The signal strength given by the adjusted volume of the bands of the respective proteins was calculated in comparison to the respective β-actin band of each sample. Significance was determined by an unpaired t-test.

To more thoroughly investigate the apoptotic potential of the three compounds, a western blot analysis was performed. Four crucial proteins involved in the process of apoptosis, namely Bax, Bcl-2, caspase 3 and PARP (poly ADP ribose polymerase) were tested (Fig. 5). Cleaved caspase 3 as well as cleaved PARP was observed in the samples incubated with 100 μM genistein in both the B164A5 and B16F10 cell lines, indicating the activation of an intrinsic or extrinsic pathway of apoptosis by proteolytic events. Neither of the three cytostatic compounds influenced the band pattern of Bcl-2 in either one of the both melanoma cell lines; however, the overall protein expression levels appeared to be slightly higher in the high metastatic B16F10 cell line compared to all samples of the B164A5 cell line. This can be also seen in the densitometric evaluation (Fig. 5C) when comparing the untreated samples of both cell lines with each other. However, the balance between pro-apoptotic Bax and anti-apoptotic Bcl-2 protein was not affected by the agents tested.

**Fig 6:**
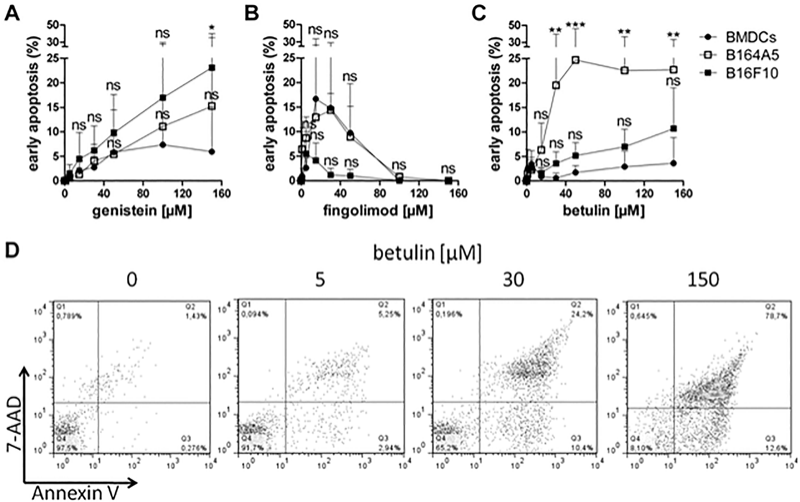
Detection of early apoptosis in B16 melanoma cells and primary dendritic cells after treatment with cytostatic drugs. Annexin V staining of B16 melanoma cells and BMDCs treated with various concentrations of genistein (A), fingolimod (B) or betulin (C) for 24 h, respectively (mean ± SD, n = 4–7). Significance was evaluated using two-way ANOVA with a Bonferroni post-test. Early apoptotic cells (Annexin V^+^/ 7-AAD^-^) were analyzed by FACS measurement. Representative dot blots of the staining are exemplarily shown for betulin in B16F10 cells (D). FACS data generated from all three compounds were used to calculate the mean values shown in the graphs.

Further investigations related to the pro-apoptotic effect of the three active agents were performed that analyzed the early signs of apoptosis by annexin V-FITC staining measured by fluorescence cytometry (Fig. 6). This assay was performed in parallel for the melanoma cells as well as the BMDCs to investigate whether there was a difference in the induction of apoptosis between strongly proliferating tumor cells and BMDCs. Genistein induced slightly more apoptosis in the B16 cells than in the BMDCs only at its highest concentration. In BMDCs we measured 6% whereas B164A5 cells revealed up to 15% or 23% in B16F10 cells, respectively. As expected, the immune modulator fingolimod caused a very high rate of apoptosis in both cell types and was already cytotoxic at low concentrations (see above; MTT assay and trypan blue staining (Fig. 1). Fingolimod seemed to act as a saponifying detergent at high concentrations; therefore, the apoptosis rate that was measurable by this type of assay apparently decreased again with concentrations of fingolimod that were above 15 μM because the cells have been physically destroyed already. Betulin triggered apoptosis in only up to 4% of BMDCs, even at the highest concentration applied. In contrast, betulin induced early apoptosis in 20% of the B164A5 cells starting from a concentration of 15 μM (p<0.01). As before, the B16F10 cells were not affected to the same extent. This gradually higher activity in melanoma cells appears to be promising with respect to the anti-cancerogenic potential of betulin, especially in the light of its immune stimulating activity described below.

### Immune modulating effects of genistein, fingolimod and betulin on dendritic cells

**Fig 7:**
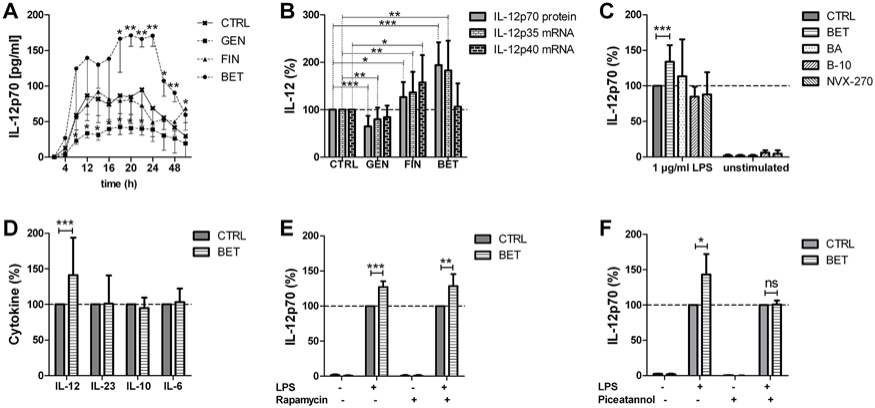
Immune response modulation of primary dendritic cells by the treatment with cytostatic drugs. Effects of 5 μM genistein (GEN), 5 μM fingolimod (FIN) or 5 μM betulin (BET), respectively, on the activation of LPS stimulated bone marrow derived dendritic cells (BMDCs). A) Released IL-12p70 was measured by an ELISA in the supernatant of LPS stimulated BMDCs for the indicated time points (mean ± SD, n = 3). B) Determination of secreted IL-12p70 protein levels 20 h after stimulation of BMDCs as well as the mRNA levels of both IL-12 subunits after an incubation period of 6 h (mean ± SD, n = 8–12). Quantitative assessment of mRNA levels was correlated to the expression of GAPDH mRNA in each sample. Significance was calculated by an unpaired t-test. C) IL-12p70 protein was detected in the supernatant of the stimulated BMDCs incubated with BET, betulinic acid (BA) and its derivatives, B-10 and NVX-270 (mean ± SD, n = 3). D) Secretion of other cytokines, such as IL-23, IL-6 and IL-10, was investigated by an ELISA of the supernatant of BET treated BMDCs (mean ± SD, n = 11). E and F) IL-12p70 protein was detected in the supernatant of LPS stimulated BMDCs incubated with BET as well as 200 ng/ml Rapamycin or 20 μM Piceatannol, respectively (mean ± SD, n = 3). For the calculations of significances, two-way ANOVA with a Bonferroni post-test was used.

To investigate the immune stimulatory potential of the cytostatic compounds, we measured the anti-tumorigenic cytokine IL-12p70 released by murine primary BMDCs (Fig. 7A). A kinetic analysis of BMDCs stimulated with 1 μg/ml LPS revealed 20 h as the preferable time point for the detection of the secreted heterodimeric protein in the supernatant and a stimulation time of 6 h as ideal for the comparison of mRNA levels of the constituting subunits (S3 Fig.). A 5 μM concentration of genistein decreased the secreted IL-12p70 protein levels of the LPS-stimulated DCs. These data were supported by the down regulation of the IL-12p35 mRNA levels (Fig. 7B). Fingolimod slightly elevated the IL-12p70 protein secretion as well as the IL-12p35 and IL-12p40 mRNA expression levels. Interestingly, betulin strongly enhanced the IL-12p70 secretion of BMDCs (p<0.001), which was accompanied and confirmed by a specific increase of the IL-12p35 subunit mRNA expression (p<0.001). In comparison, very closely related compounds, such as betulinic acid and semi-synthetic glycosylated derivatives of betulinic acid, called B-10 and NVX-270, which are highly cytotoxic in cancer cells [30], failed to raise the secretion of IL-12p70 of the LPS-stimulated BMDCs (Fig. 7C). Nevertheless, the release of other cytokines, e.g., IL-6, IL-10 or IL-23, was not changed in the betulin-treated BMDCs after 20 h (Fig. 7D). Moreover, costimulation with Rapamycin which inhibits the mTOR-dependent pathway via MyD88 signaling cascade did not influence the activity of betulin. In contrast, additional treatment with Piceatannol which inhibit the NF-κB and JAK-1/STAT signaling [31] abolished the effect of betulin to increase IL-12p70. This led us to the conclusion that betulin seems to target the NFκB-dependent pathway to IL-12 but the signaling cascade of betulin still needs to be further investigated.

### Immune modulating effects of genistein, fingolimod and betulin on T cells

**Fig 8:**
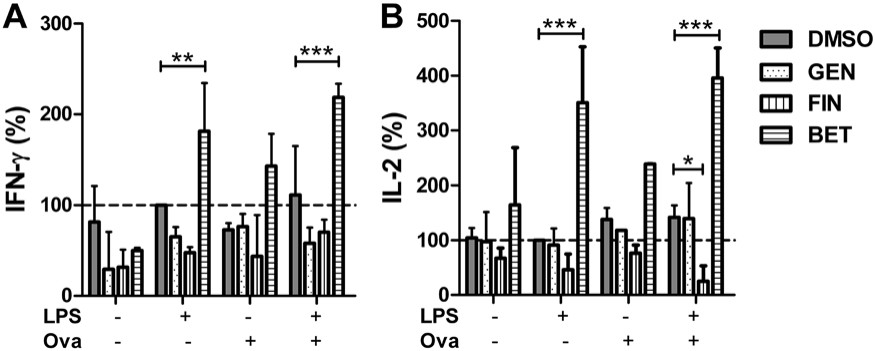
Immune response modulation of antigen specific T cells by the treatment with cytostatic drugs. The spleen cells of OT-I mice were stimulated with 1 μg/ml LPS and 13.5 μg/ml ovalbumin (OVA) as the antigen and additionally incubated with 5 μM genistein (GEN), 5 μM fingolimod (FIN) or 5 μM betulin (BET), respectively, for 44 h (mean ± SD, n = 3). The concentration of IFN-γ (A) and IL-2 (B) in the supernatant of the cells was measured by an ELISA. Significance was calculated using two-way ANOVA with a Bonferroni post-test.

The immune stimulating potential regarding the dendritic cell activity was promising, especially of betulin. Thus, we further sought to determine whether the modified activation of the stimulated dendritic cells by these three compounds is transmitted to an altered activation of anti-cancerogenic, cytotoxic T cells. Therefore, we used spleen cells of OT I mice as a model system expressing the ovalbumin-specific transgenic T cell receptors. The T cell activity was determined by IFN-γ and IL-2 cytokine detection in the supernatant (Fig. 8). Co-cultivation of the stimulated spleen cells with genistein had no effect on the cytokine production of T cells compared to the DMSO treated cells serving as the control. Fingolimod slightly down-regulated the IL-2 protein levels in the supernatant (p<0.05). In contrast, betulin strongly elevated the IFN-γ as well as the IL-2 production of stimulated spleen cells, indicating an increased activation of CD8^+^ ovalbumin-specific T cells (p<0.001).

### Cytotoxic effect of betulin in an antigen-specific B16OVA model system

**Fig 9:**
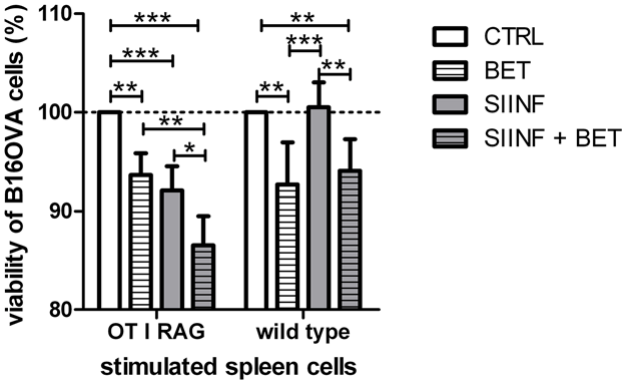
Cytotoxic effect of betulin in an antigen-specific B16OVA model system. B16OVA cell viability after treatment with prestimulated spleen cells (mean ± SD, n = 4). 1x10^5^ B16OVA cells were treated with 3.2x10^6^ spleen cells, isolated of OT I RAG mice or WT mice, respectively. The CD8^+^ OVA TCR^+^ effector T cell to B16OVA target cell ratio for OT I RAG cells is 7:1. The viability of the CFSE stained B16OVA cells was analyzed by FACS measurements. Significance was calculated using two-way ANOVA with a Bonferroni post-test.

We measured the B16OVA killing potential of betulin treated spleen cells in a SIINFEKL-specific model system (Fig. 9). Betulin treatment alone induced cell death of B16OVA cells (p ≤ 0.01). The antigen-specific loading of DCs with SIINFEKL peptide alone led to the recognition and destruction of SIINFEKL-carrying B16OVA cells by CD8^+^ OVA TCR^+^ cytotoxic T cells (p ≤ 0.001). However, the combination of both decreased the viability of B16OVA cells in an even higher extend (p ≤ 0.001). Interestingly, the loading process of the peptides on MHC I molecules (S4A Fig.) as well the frequency of SIINFEKL-specific T cell receptors (S4B Fig.) is not influenced by betulin.

## Discussion

In this investigation both the cytotoxic actions of genistein, fingolimod and betulin on melanoma cells and their immune modulating effects on dendritic cells and T cells were studied side-by-side in parallel. Interestingly, our studies revealed new actions on malignant cells. But prominently, our data indicated that these compounds have highly differential effects on immune cells. Genistein exhibited toxic properties typically expected from a cytostatic drug, while fingolimod’s action at low micromolar concentrations seemed to be due to its amphiphilic properties. In contrast, betulin showed both simultaneous and effective anti-melanoma and immune-stimulating activity, qualifying this compound for an integrated tumor cytotoxic and cellular immune therapy.

Our experiments revealed an antiproliferative function of genistein in B16 melanoma cells by a G2/M arrest of the cell cycle and by the induction of apoptosis. Interestingly, at higher concentrations genistein had a stronger pro-apoptotic effect in the more rapidly dividing B16 cells than in BMDCs, which is supposed to be typical for a chemotherapeutic drug. Genistein most likely prevented the metastatic activity of the B16F10 cells by the inhibition of matrix metalloproteinases [19]. Mahmoud *et al*. have reviewed the antiproliferative effects of genistein in prostate cancer, which is explainable amongst others by the inhibition of tyrosine kinases or the inhibition of NF-κB, respectively. The inhibition of NF-κB fits particularly well with other reports about genistein’s anti-inflammatory properties. Our experiments demonstrated that 5 μM of genistein decreased the LPS-induced IL-12p70 secretion of BMDCs. This has not been shown before, but in principle the immune suppressive effects of cytostatics are accepted despite the expected disadvantage for the long-term cancer prognosis. The production of IL-12p70, as the most prominent cytokine with an anticancer activity, depends on simultaneous IL-12p35 and IL-12p40 gene expression and their proper assembly the IL-12p70 heterodimer. Of note, the transcription of these IL-12 subunits is NF-κB/c-Rel and NF-κB/RelA-dependent, respectively [8]. Taken this into account the significant down-regulation of IL-12p35 at mRNA levels indicates that genistein may inhibit NF-κB/c-Rel. Other publications suggested that genistein suppressed TLR4 up-regulation [32], which would also explain the reduced IL-12p70 production in our experiments because we used a TLR4 ligand for stimulation of the BMDCs. In agreement with our data, Wei *et al*. showed in human monocyte-derived DCs that genistein treatment suppressed the LPS-induced IL-12 secretion [33]. The activation of the T cells measured by IL-2 and IFN-γ secretion of splenocytes co-stimulated with genistein was also slightly decreased in our experiments, although the results failed to reach significance. Taken together, despite the fact that genistein is already in clinical trials for different types of cancer, in our *in vitro* system with melanocytes, genistein, at an equimolar level, had a lower antiproliferative activity compared to the other two compounds tested. Additionally, genistein demonstrated unfavorable immunosuppressive characteristics by decreasing the IL-12p70 secretion of dendritic cells. This, in the light of our aim of a dual approach for a future cancer therapy disqualifies genistein.

In all of the assays performed, fingolimod exhibited a very strong cytotoxic effect on B16 melanoma cells already starting at the lowest concentration tested. Fingolimod stopped B16 cell proliferation by blocking cells during the G0/G1 phase of the cell cycle which can be explained by the fingolimod-induced activation of protein phosphatase 2A [34]. Recently, it was discovered that fingolimod inhibited sphingosine kinase 1 (SK1), which may be relevant to cancer progression [35]. In our laboratory, Schröder *et al*. showed that stimulation of SK1^-/-^ DCs produced more IL-12p70 than wild-type DCs [36], which coincides with our results showing that fingolimod-treated DCs have increased IL-12p70 levels due to an enhanced mRNA expression of both subunits. While reaching toxic ranges Permpongkosol *et al*. described that 40 μM of fingolimod caused the activation of p38 MAPK in DU145 cells, which would also explain the anti-tumorigenic effect on the one hand and the IL-12 increasing effect of fingolimod on the other hand [37]. In contrast, Zeng *et al*. found a down-regulation of IL-12 secretion of BMDCs that had been treated with LPS and 0.5 μM fingolimod [38]. However, our experiments revealed a decreased IL-2 release in treated splenocytes, which was probably due to the enhanced generation and function of regulatory T cells and the inhibition of pro-inflammatory Th1 cells by fingolimod [39,40]. Thus, an enhancement of the immune reaction failed. In conclusion, fingolimod would not be an optimal anti-melanoma drug because of its high cytotoxicity that has been demonstrated in BMDCs. We need to assume that healthy tissue cells can also be destroyed by this compound. Furthermore, a few cases of skin cancer have been already reported during the treatment of multiple sclerosis with fingolimod (http://www.webmd.com/multiple-sclerosis/news/20080416/good-news-for-oral-ms-drug-fingolimod).

The third and most promising substance we have investigated here is betulin. This triterpen exhibited the highest cytostatic activity, presenting IC_50_ values close to 2 μM for both of the B16 sub cell lines. Already after a short incubation time in the MTT assay, indicating a blockade of cellular respiration. One possible reason for this activity may be a decrease in CYP2E1, which was shown in acute ethanol-induced fatty liver by betulin administration [13]. Other possible reasons include a triggered cytochrome c release [41] or targeted mitochondrial apoptosis [42], which has previously been shown for betulinic acid (BA). In our study, betulin showed delayed antiproliferative effects and significantly stronger pro-apoptotic effects in B16 cells than in BMDCs, even at relatively low concentrations. This indicates that betulin is more cytotoxic in rapidly dividing cells, which would be a preferred property of a chemotherapeutic drug. Other groups have proven that betulin induced programmed cell death in human cancer cells [43,44]. Additionally, it has been reported in the literature that betulin’s carboxylated form, BA, and the derivatives of BA are even stronger active agents against human melanoma cells [23,42,45]. We investigated the immune modulating function of the betulin related compounds but the derivatives failed to influence the activation of BMDCs as measured by IL-12p70 release. In contrast, we found for the first time that 5 μM betulin potently increased the secretion of active heterodimeric IL-12 of the stimulated BMDCs and the corresponding mRNA level of the p35 subunit. Although Wan *et al*. found a decrease of TLR4 expression by betulin in ethanol-stimulated hepatic stellate cells [13], it is not yet known how betulin directly interacts in the TLR4 signaling of BMDCs. However, the carboxylation of betulin to BA obviously prevented the ability to interact in the signaling pathway. Given that the increase of IL-12 in betulin-treated samples was due to an elevated IL-12p35 mRNA level, indicates that betulin may enhanced the NF-κB/c-Rel-dependent signaling, whereas NF-κB/RelA was not affected [8]. A dependency of NF-κB in the signaling pathway of betulin is strengthen by the costimulation of BMDCs with Picetannol which abolished the IL-12 increasing effect of betulin. These findings are in line with Kasperczyk *et al*. who described that BA activated p50 and p65 NF-κB subunits [46] but are in contrast to the published data that suggests that betulin inhibits NF-κB signaling [26]. However, betulin also elevated the IFN-γ and IL-2 secretion of stimulated spleen cells in our hands. These findings led to the conclusion that the increased activation of DCs by betulin is transmitted to an increased activation of T cells. Finally, we could prove in an OVA-specific model system that the presence of betulin alone led to an intensified elimination of B16OVA cells. These killing is in the same range as an antigen-specific priming of DCs with SIINFEKL peptide which led to the activation of OVA TCR-specific CD8^+^ T cells that than recognize and kill the B16OVA cells. The combination of the antigen specific DC loading and the treatment with betulin decreased the B16 cell viability even more. Interestingly, the frequency of SIINFEKL-presenting dendritic cells as well as the amount of SIINFEKL-recognizing CD8^+^ T cells was not affected by betulin. Therefore, we conclude that the decreased viability of B16 cells is due to an enhanced activity of the immune cells by betulin without affecting the amount of DCs or CTLs. Another reason for the decreased B16 viability is the cytotoxic potential of betulin on melanoma cells which we proved already in other assays. The efficiency of betulin may overcome the immunosuppressive environment of a tumor *in vivo*. For that reason, the influence of betulin in the signaling of immune cells is worth further investigation.

## Conclusion

With this investigation, we presented experimental evidence that cytostatic drugs are not necessarily immune suppressive. Our new findings regarding the actions of genistein, fingolimod and betulin on B16 cell lines complete the data in the literature regarding their antiproliferative and pro-apoptotic effects on cancerous cell lines. From an *in vitro* point of view, all three candidates presented antiproliferative and pro-apoptotic effects on the B16F10 and B164A5 murine melanoma cell lines. These findings are of real interest for the medico-pharmaceutical field because they present large dose-response curves for these events. However, betulin exhibits features of an immune stimulating cancer drug that increased DC and T cell activity, whereas fingolimod stimulated DCs only, and genistein suppressed DC activation. Therefore, betulin may be useful as an adjuvant in cancer therapy by enhancing the magnitude, quality and longevity of specific immune responses to antigens and has a moderate toxicity in immune cells. Because cytokine immunotherapy is a very promising mainstay of treatment for malignant melanoma, we also hypothesize that betulin may modulate the immune cell expression of IFN-γ and cytotoxic T cell effector mechanisms *in vivo*, which needs to be tested in the near future. This immunotherapy would be even more effective if the dendritic cells of the individual patient get additionally loaded with specific melanoma antigens to force the immune response directly against the tumor tissue. Thus, this investigation may foster both the development of betulin-derived structural analogs and—as a more general approach—a new method of screening strategies for compounds combining cytostatic and immune stimulating properties.

## Supporting Information

S1 Fig Overlay graphs of MTT assay and apoptosis measurement. Overlay graphs of the data obtained from MTT assay and Annexin V/7-AAD staining shown representatively for the B16F10 cell line. Arrows indicate the chosen concentration of each substance (A: genistein, B: fingolimod, C: betulin) in which the antiproliferative activity is quite high (of around 70%) and the apoptotic potential is relatively low.
(TIF)

S2 Fig PI staining of B16 melanoma cells after treatment with cytostatic drugs. Representative images of B164A5 (A–D) and B16 F10 (E–H) cells correlated to the different phases of the cell cycle (green: G0/G1; yellow: S; blue: G2/M) analyzed by PI staining after treatment with DMSO (A, E), 100 μM genistein (GEN: B, F), 5 μM fingolimod (FIN: C, G) or 5 μM betulin (BET: D, H) for 24 h, respectively.
(TIF)

S3 Fig IL-12 mRNA level over time of LPS stimulated dendritic cells. Time kinetics to determine the optimal time point for measuring the mRNA expression of LPS stimulated BMDCs (mean ± SD, n = 3) co-stimulated with 5 μM genistein (GEN), 5 μM fingolimod (FIN) or 5 μM betulin (BET), respectively. RNA was isolated after several time points and reverse transcribed into cDNA. This cDNA was used as a template to perform real-time PCR with TaqMan probes for IL-12p35 (a) and IL-12p40 (b) correlated to GAPDH mRNA as a control housekeeping gene. Significance was calculated using two-way ANOVA with a Bonferroni post-test. We decided to use 6 h of stimulation for further investigations (Fig. 7B).
(TIF)

S4 Fig Determination of H2kb molecules loaded with SIINFEKL peptide as well as the detection of CD8^+^ OVA TCR^+^ T cells in OT I RAG and WT spleen cells. A) Amount of dendritic cells loaded SIINFEKL peptide on H2kb molecules after pulsing of spleen cells with 1 μg/ml SIINFEKL and 50 Units/ml IL-2 for 24 h. B) Amount of CD8^+^ T cells expressing SIINFEKL specific TCR (OVA TCR) at their surface of spleen cells isolated of OT I RAG or wild type mice, respectively.
(TIF)
