# Supplementary figures and images for: Simultaneous and Dose Dependent Melanoma Cytotoxic and Immune Stimulatory Activity of Betulin
Source: PLoS One. 2015 Mar 10;10(3):e0118802. doi: 10.1371/journal.pone.0118802 (PMC4355578; doi:10.1371/journal.pone.0118802)

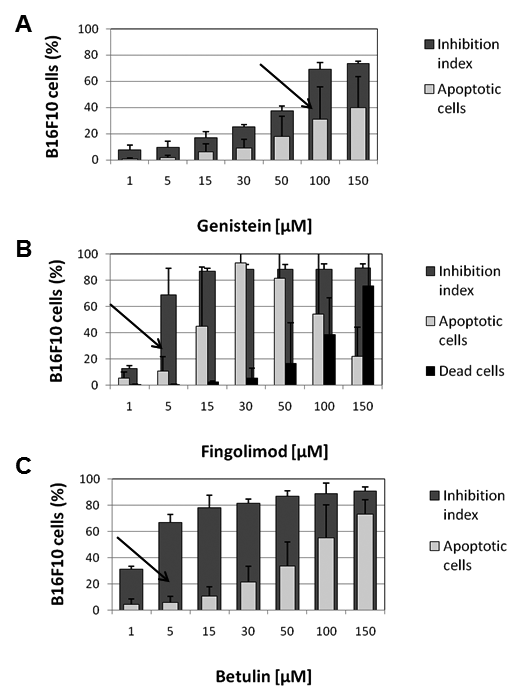

Supplement: S1 Fig — Overlay graphs of the data obtained from MTT assay and Annexin V/7-AAD staining shown representatively for the B16F10 cell line. Arrows indicate the chosen concentration of each substance (A: genistein, B: fingolimod, C: betulin) in which the antiproliferative activity is quite high (of around 70%) and the apoptotic potential is relatively low. (TIF) [file pone.0118802.s001.tif]

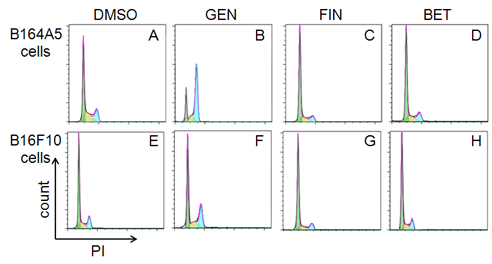

Supplement: S2 Fig — Representative images of B164A5 (A–D) and B16 F10 (E–H) cells correlated to the different phases of the cell cycle (green: G0/G1; yellow: S; blue: G2/M) analyzed by PI staining after treatment with DMSO (A, E), 100 μM genistein (GEN: B, F), 5 μM fingolimod (FIN: C, G) or 5 μM betulin (BET: D, H) for 24 h, respectively. (TIF) [file pone.0118802.s002.tif]

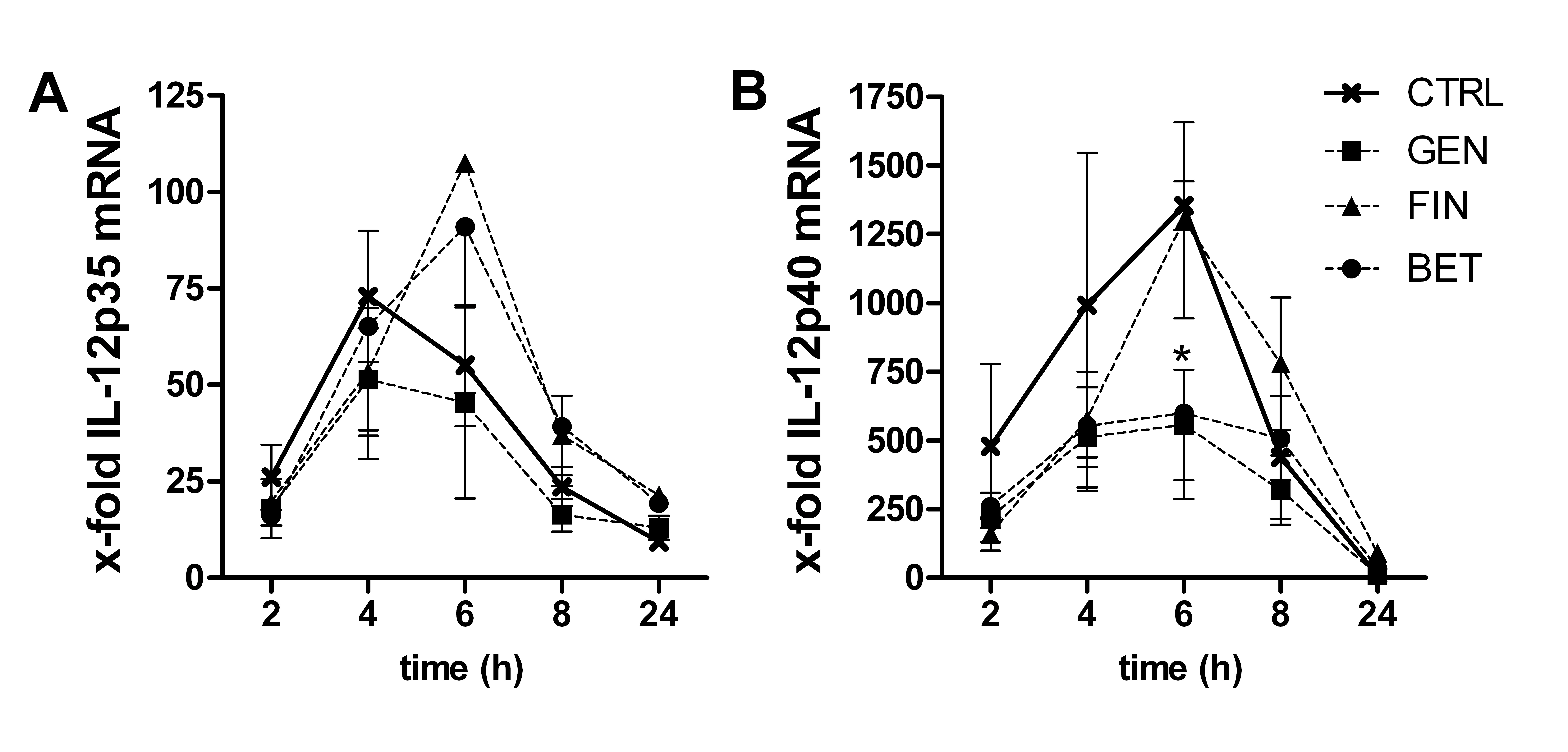

Supplement: S3 Fig — Time kinetics to determine the optimal time point for measuring the mRNA expression of LPS stimulated BMDCs (mean ± SD, n = 3) co-stimulated with 5 μM genistein (GEN), 5 μM fingolimod (FIN) or 5 μM betulin (BET), respectively. RNA was isolated after several time points and reverse transcribed into cDNA. This cDNA was used as a template to perform real-time PCR with TaqMan probes for IL-12p35 (a) and IL-12p40 (b) correlated to GAPDH mRNA as a control housekeeping gene. Significance was calculated using two-way ANOVA with a Bonferroni post-test. We decided to use 6 h of stimulation for further investigations (Fig. 7B). (TIF) [file pone.0118802.s003.tif]

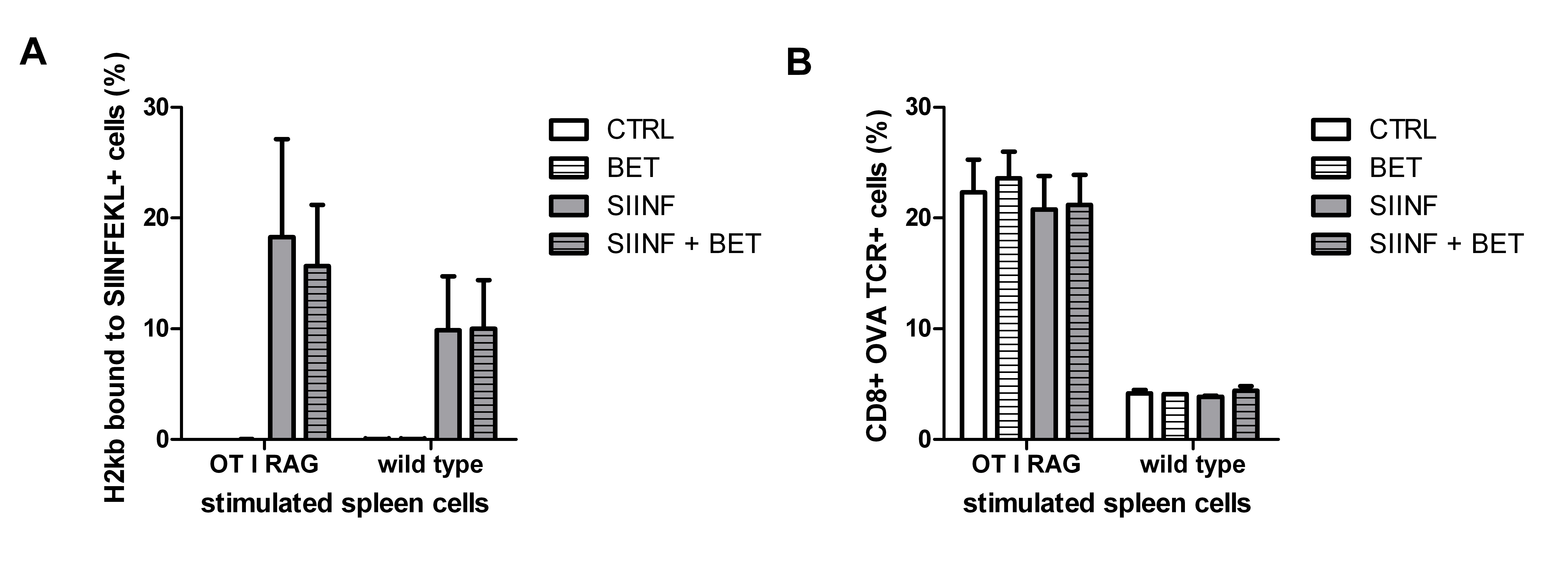

Supplement: S4 Fig — A) Amount of dendritic cells loaded SIINFEKL peptide on H2kb molecules after pulsing of spleen cells with 1 μg/ml SIINFEKL and 50 Units/ml IL-2 for 24 h. B) Amount of CD8+ T cells expressing SIINFEKL specific TCR (OVA TCR) at their surface of spleen cells isolated of OT I RAG or wild type mice, respectively. (TIF) [file pone.0118802.s004.tif]
